# A Case of Hairy Cell Leukemia Variant: Literature Analysis With Focus on Unmet Needs

**DOI:** 10.7759/cureus.47085

**Published:** 2023-10-15

**Authors:** Amitabh Kumar Upadhyay, Manoj Kumar, Anil Prasad, Shashank Shekhar, Reetal Singh

**Affiliations:** 1 Medical Oncology, Tata Main Hospital, Jamshedpur, IND; 2 Pathology, Tata Main Hospital, Jamshedpur, IND; 3 Medical Oncology, Meherbai Tata Memorial Hospital, Jamshedpur, IND

**Keywords:** cladribine, massive splenomegaly, lymphoma, hairy cell leukaemia, hairy cell leukemia variant

## Abstract

Hairy cell leukemia variant (HCLv) is a sporadic, B-cell non-Hodgkin lymphoma classified under chronic lymphoproliferative disorders. HCLv usually presents with easy fatigue, dragging pain abdomen, anemia, splenomegaly, hepatomegaly, initially leukocytosis followed by leucopenia, hairy cells in the smear and bone marrow, and an increased risk of infections. There is hypercellular bone marrow, and cytopenias are secondary to hypersplenism. It is essential to differentiate HCL from disorders like classic hairy cell leukemia (HCLc), splenic marginal zone lymphoma, and splenic diffuse red pulp lymphoma, as these are biologically different, with divergent approaches and outcomes. HCLv is poorly responsive or primary refractory to standard purine analogs cladribine or pentostatin. It has lower response rates to even cladribine and rituximab combination, a standard of care for classic HCL with very good response rates. Here, we present a case of an elderly male who presented with splenomegaly and leukocytosis, diagnosed as HCLv, and was treated with a cladribine and rituximab-based regime but showed residual cells in bone marrow on flow cytometry at six months post-treatment. There were no residual cells in peripheral blood in flow cytometry. Various aspects of the disease are discussed here with a detailed literature analysis. There is a definite unmet need for research on better treatment options in HCLv to improve its overall outcome.

## Introduction

Hairy cell leukemia (HCL) is a sporadic, indolent, B-cell non-Hodgkin lymphoma (NHL) that is classified under chronic lymphoproliferative disorders and comprises classical HCL (HCLc) and HCL-like disorders [1,2]. Hairy cell leukemia variant (HCLv) is a provisional entity in the most recent revision of the WHO 2016 classification [1,2]. It presents with anemia, thrombocytopenia, splenomegaly, hepatomegaly, and leukocytosis initially followed by leucopenia, increased risk of infections, and easy fatigue. It is essential to differentiate HCL from HCL-like disorders as these are biologically different, with divergent treatment approaches and prognoses. Morphologically, these disorders look similar, and immunophenotyping helps to distinguish between these entities. In 2008, the World Health Organization (WHO) reclassified the disorders into separate categories of lymphoproliferative disorders [3]. The four immunophenotyping markers which help in differentiation are CD11C, CD103, CD25, and CD123. Each marker is assigned a score of 1 if expressed and 0 if not expressed. Classical HCL scores 3 to 4, whereas the HCL-like disorders have low immunological scores of 0 to 1 [1-3]. HCLv is a mature lymphoid B-cell disorder characterized by the identification of hairy cells, a specific immunophenotypic profile, and a different clinical course than other disorders of a similar spectrum like HCLc, splenic marginal zone lymphoma (SMZL), and splenic diffuse red pulp lymphoma (SDRPL). HCLv patients do present with splenomegaly, leukocytosis, or infections. The circulating lymphoid cells have a morphology intermediate between prolymphocytes and hairy cells. The HCL immunological score is low (0 or 1), with no expression of CD25 and inconstant or weak CD123 expression [1-3]. 

HCLv is a more aggressive disease than HCLc, with a different clinical course and usually poorly responsive or primary refractory to standard treatment options. The response rate with purine analogs cladribine or pentostatin is greater than 90% for HCLc but in the 40-50% range for HCLv, with most responses being partial [4]. In HCLv, the abnormal lymphoid cells do not usually express CD25, CD200, and CD123. The proto-oncogene B-Raf (BRAFV600E) gene mutation is not present in HCLv but is a hallmark of HCLc [1-4]. Similarly, mitogen-activated protein kinase kinase 1(MAP2K1) gene mutations are present in about 30% of HCLv cases [1-4]. HCLv is treated with more aggressive therapies with purine analogs and rituximab. The clinical course of HCLv is more aggressive, with a median overall survival (OS) of 9 years [1-4]. Here, we present a case of HCLv who presented with splenomegaly and leukocytosis and was treated with a cladribine and rituximab-based regime. 

## Case presentation

**Figure 1:**
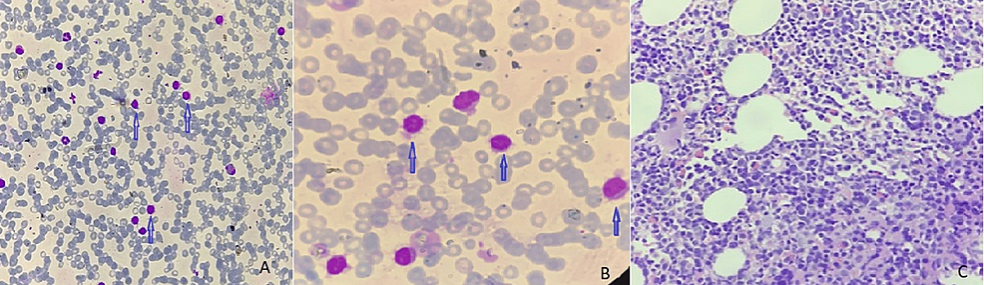
(A) Peripheral smear Leishman staining in 100X magnification showing atypical lymphoid cells with fine hairy processes (blue arrow); (B) bone marrow aspiration Leishman 400X magnification showing hairy cells having round to oval nucleus with moderate pale grey cytoplasm (blue arrow); (C) bone marrow biopsy hematoxylin and eosin (H&E) staining in 100X magnification showing cellular marrow, medium-sized lymphoid cells with oval nuclei, open chromatin, absent nucleoli, and a characteristic serrated cytoplasmic border

A 68-year-old male patient with Eastern Cooperative Oncology Group (ECOG) performance status of one presented with complaints of heaviness in the left upper side of the abdomen for one year, which was gradually progressive and worsened in the last four months. He also had B symptoms of fever and significant weight loss of more than 10% in the previous six months. His physical examination revealed massive splenomegaly, palpable 3 cm below the umbilicus. His hemogram showed a white blood cell (WBC) count at 25.8 x109/L, hemoglobin (Hb) level at 10.1 gm/dl, and platelet (PLT) count at 92 x 109/L. Differential counts showed an absolute neutrophil count of 1290 (N), 23994 lymphocytes (L), 516 monocytes (M), no eosinophils (E), and no basophils (B). Peripheral smear showed atypical lymphoid cells, round to oval nuclei with exemplary hairy processes, which are larger than the size of mature lymphocytes (Figure 1A). His bone marrow aspiration showed diluted marrow with suppression of erythroid and myeloid series, proliferation of lymphoid cells, mostly atypical lymphoid cells (68%), and absolute lymphocytosis. Lymphoid series accounted for 82% of all nucleated cells (Figure 1B). Bone marrow biopsy showed bony trabeculae with normocellular marrow, trilineage differentiation with predominantly normoblastic to micronormoblastic erythropoiesis, normal myelopoiesis, and adequate megakaryocytes. Focal marrow areas showed lymphoid cell clusters, intermediate size with coarse chromatin and perinuclear halo. There was no evidence of granulomatous inflammation (Figure 1C). Flow cytometry was done on bone marrow aspirate and showed negative for CD5, CD10, CD25, CD123, and CD200 and positive for CD103 and CD11c, suggesting the variant form of hairy cell leukemia. B-RAF mutation analysis by polymerase chain reaction (PCR) was negative. Contrast-enhanced computed tomography (CECT) of the neck, thorax, and abdomen revealed no significant abnormality or lymphadenopathy in the neck or thorax region. There was gross splenomegaly (21 cm) and hepatomegaly (19 cm). 

**Figure 2:**
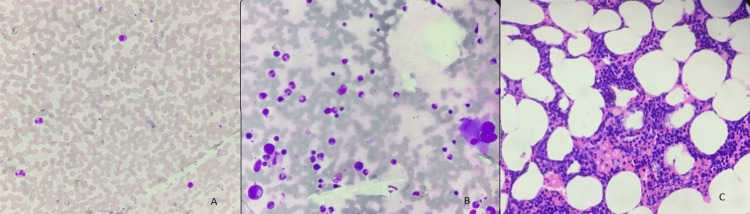
(A) Peripheral smear Leishman staining in 10X magnification showing normal leukocyte count and distribution, (B) bone marrow aspiration Leishman 40X magnification showing normal hematopoietic cells and lymphocytes, and (C) bone marrow biopsy post-chemotherapy Leishman H&E 10X magnification showing remission of hairy cell leukemia

**Figure 3:**
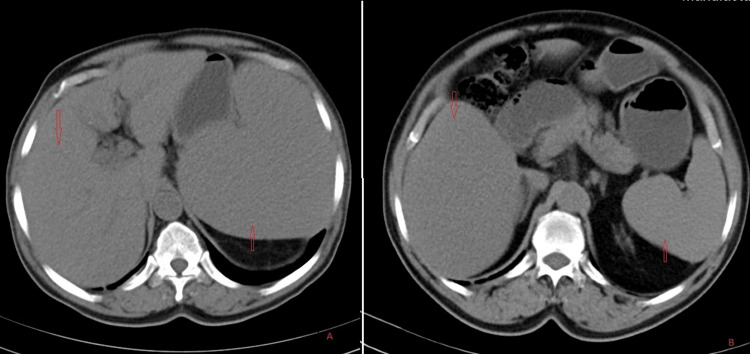
(A) Axial plane of CECT abdomen showing the presence of splenomegaly (upward red arrow) and hepatomegaly (downward red arrow) and (B) axial plane of CECT abdomen at six months after treatment showing significant resolution of splenomegaly (upward red arrow) and hepatomegaly (downward red arrow) CECT: Contrast-enhanced computerized tomography

**Figure 4:**
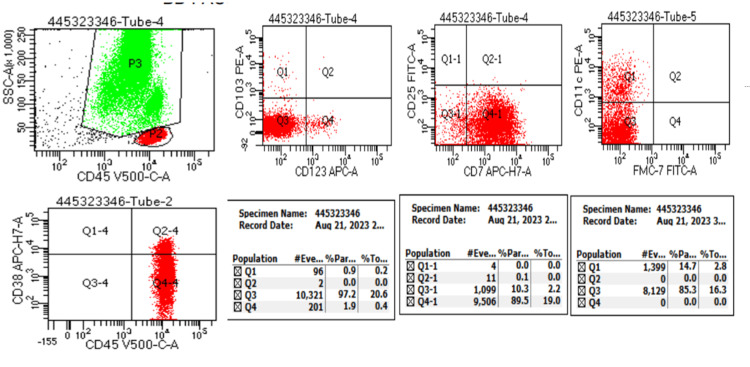
Bone marrow flow cytometric findings show 0.5% B lymphoid cells with positive expression of B cell markers (CD103, CD11c, and CD20)

**Figure 5:**
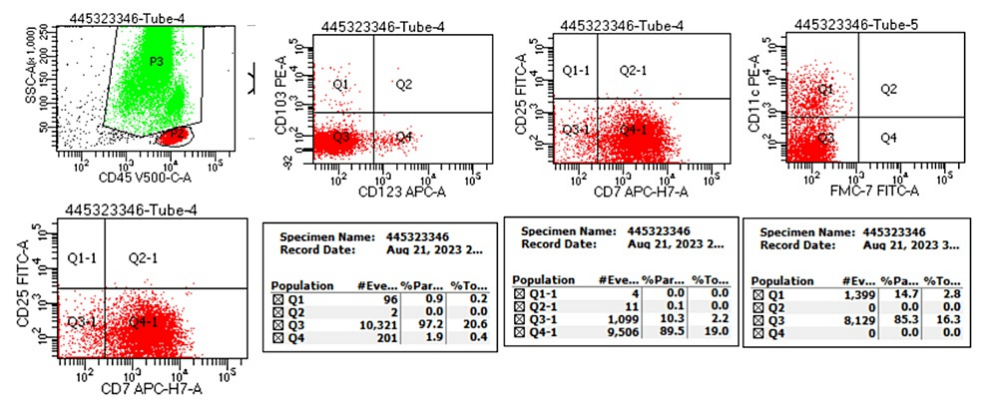
Peripheral blood flow cytometric findings identify 21% lymphocytes (16% of total events are T cells, <1% B cells and 4% NK cells), and hairy cell markers are negative NK: Natural killer

This patient received systemic therapy with one cycle of cladribine in a dose of 0.15 mg/Kg on days 1-5, with eight weekly doses of rituximab 375 mg/m2 beginning day one. The hemogram findings during treatment and at six months are shown in Table 1. Follow-up hemogram at six months showed a WBC count of 5.4 x109/L, a Hb level of 12.9 gm/dl, and a platelet count of 105 x 109/L. Differential counts showed 71% neutrophils (N), 17% lymphocytes (L), 9% monocytes (M), 03% eosinophils (E), and 00% basophils (B). Peripheral smear showed normocytic normochromic RBC (Figure 2A). Marrow aspiration findings showed cellular reactive marrow with mild erythroid hyperplasia, lymphoid series accounting for 12%, and no evidence of granulomas/residual malignancy (Figure 2B). Bone marrow biopsy findings showed bony trabeculae with normocellular marrow, trilineage differentiation with predominantly normoblastic to micronormoblastic erythropoiesis, normal myelopoiesis, and adequate megakaryocytes. Focal areas (1%) show lymphoid cell clusters, intermediate size with coarse chromatin, and perinuclear halo without any evidence of granulomatous inflammation (Figure 2C). CECT Abdomen shows hepatomegaly and splenomegaly (12.36cm) with standard shape, density, and enhancement. The splenomegaly had markedly reduced after treatment (Figures 3A, 3B). Flow cytometry revealed 0.5% B lymphoid cells on bone marrow aspirate with positive expression of hairy cell markers (CD103+CD11c+ CD20+ bright) (Figure 4). Flow cytometry identified 21% normal lymphocytes in peripheral blood consisting of 16% T cells, 4% NK cells, and 1% B cells. There was no evidence of any residual cells with hairy cell markers (Figure 5). 

**Table 1:** Hemogram at the time of chemotherapy and rituximab doses and at six months

Drugs	WBC	Hb (g/dL)	Platelets	N (%)	L (%)	M (%)	E (%)	B (%)
Cladribine (Day1 to Day 5), Rituximab Week 1	25.8 x 10^9^/L	10.1	92 x 10^9^/L	5	93	2	0	0
Rituximab - Week 2	8.9 x 10^9^/L	09.5	91 x 10^9^/L	18	78	4	0	0
Rituximab - Week 3	2.7 x 10^9^/L	13.2	71 x 10^9^/L	26	65	8	1	0
Rituximab - Week 4	6.5 x 10^9^/L	13.8	98 x 10^9^/L	62	31	6	1	0
Rituximab - Week 5	4.8 x 10^9^/L	12.1	73 x 10^9^/L	79	11	6	3	1
Rituximab - Week 6	4.7 x 10^9^/L	12.8	78 x 10^9^/L	73	15	7	5	0
Rituximab - Week 7	5.4 x 10^9^/L	12.4	115 x 10^9^/L	72	14	8	6	1
Rituximab - Week 8	4.7 x 10^9^/L	12.3	110 x 10^9^/L	73	17	8	2	0
At six months	5.4 x 10^9^/L	12.9	105 x 10^9^/L	71	17	9	3	0
Normal reference range	4.0-11.0 x 10^9^/L	11.5-16.5 g/dL	150-400 x 10^9^/L	60-70%	30-40%	2-8%	1-6%	0-1%

The patient became asymptomatic at six months. Due to discrepant results for residual cells in the bone marrow and peripheral blood, he has been counseled for a second course of rituximab for eight doses to have undetected residual cells in the bone marrow. 

## Discussion

HCL-v, first described by Cawley et al. [5], occurs at an incidence of 0.03 per 100,000 persons per year, has a male predominance (ratio of 6:1), and constitutes around 0.4% of all chronic lymphoid malignancies [1-5]. HCL-v is a disease of the elderly, with a median age of 71 [1-4]. Initial symptoms are abdominal discomfort or pain, usually secondary to splenomegaly, hepatomegaly, and symptoms derived from cytopenias such as anemia, bleeding, and/or recurrent infections. Usually, diagnosis is delayed for many years due to nonspecific symptoms of weakness, easy fatigue, and dragging pain in the abdomen, which is attributed to advanced age. Sometimes, we find a history of blood transfusions because of anemia. The diagnosis warrants a detailed clinical history, physical examination, and investigations, including peripheral blood smear, bone marrow morphology, immunophenotyping, and molecular diagnostics. Peripheral blood morphology of HCLv typically shows small-to-medium lymphocytes with circumferential cytoplasmic projections. Patients usually present with massive splenomegaly, leukocytosis without monocytopenia, and a hypercellular bone marrow that can be easily aspirated. Patients develop cytopenias with time because of hypersplenism [1-4]. 

SMZL with villous lymphocytes is another indolent B-cell lymphoma that may be mistaken for HCL. However, there are unipolar or bipolar cytoplasmic projections rather than circumferential projections, as seen in HCL [1-4]. SMZL is characterized by abnormal lymphoid cells with round nuclei, condensed chromatin, and basophilic cytoplasm with polar short villi (villous lymphocytes) in the peripheral blood [1-4]. SDRPL is a provisional entity, very close if not identical to HCL-V. SDRPL could be the first step before the occurrence of HCLv and is characterized by the presence of a large proportion of small to medium-sized villous lymphoid cells in peripheral blood [1-4]. The abnormal lymphoid cells have a polar distribution of the villi, and the nucleolus is small or invisible. The characteristics of HCLc, HCLv, SMZL, and SDRPL are further described in Table 2 [1-4].

**Table 2:** Analysis of disease characteristics of HCLc, HCLv, SMZL, and SDRPL TRAP: Tartrate-resistant acid phosphatase, IGHV: immunoglobulin heavy chain, CCND3: cyclin D3, del: deletion; HCLc: classic hairy cell leukemia; HCLv: hairy cell leukemia variant; SMZL: splenic marginal zone lymphoma; SDRPL: splenic diffuse red pulp lymphoma Data from [1-4] Table creation: AK Upadhyay

-	HCLc	HCLv	SMZL	SDRPL
B symptoms	Rare	Rare	Approx 1/4th	Approx 1/3rd
Blood smear	Reniform or oval nuclei, circumferential long villi, inconspicuous nucleoli	Abundant circumferential villi and prominent nucleoli	Polar villi and inconspicuous nucleoli	Broad-based polar cytoplasmic extensions
Leukocyte count	Low	High	High	High
Median age	59	71	69	66.5
TRAP activity	Positive	Negative	Weak	Negative
Surface Immunoglobulin	IgM	IgG	IgM	Biclonal or monoclonal
Immunophenotype	CD11c+, CD25+, CD123+, CD 200+	CD11c+, CD25-, CD123-, CD200-, CD103+	CD11c+, CD25±, CD103-	CD11c-, CD25-, CD123-, CD103-, DBA.44+
Annexin A1	Positive	Negative	Negative	Negative
Cytogenetics	del 13q, del 7q	del 17p	del 7q, trisomy 3	del 7q, trisomy 18, del 17p
IGHV mutation	Mutated	Unmutated	Usually mutated	Positive
BRAF mutation	Mutated	Unmutated	Not reported	Negative
MAP2K1 positivity	Less common	More common	Not reported	Positive
CCND3	Wild type	Mutated	Unreported	Mutated
Spleen involvement	Red pulp	Red pulp	White pulp	Red pulp
Bone marrow infiltration	Inter-sinusoidal	Mostly intra-sinusoidal, rarely inter-sinusoidal	Paratrabecular and predominantly inter-sinusoidal	Interstitial, rarely nodular
Treatment	Purine analogs, rituximab	Purine analogs and rituximab, Splenectomy	Splenectomy, rituximab	Splenectomy
Median overall survival from diagnosis (years)	20	9	10	5-year survival of more than 90%

HCLv is inherently aggressive and does not respond well to single-agent purine analogs. Cladribine given as a single five-day daily, the two-hour intravenous infusion has shown poorer response rates than HCLc [1-4]. The combination therapy of purine analogs with an anti-CD20 monoclonal antibody, rituximab, is the first-line treatment option for HCLv [6]. In the largest phase 2 study by Chihara et al., 20 patients of HCLv received cladribine 0.15 mg/kg on days one to five with eight rituximab doses of 375 mg/m^2^ at weekly intervals, beginning day one [6]. Patients were eligible for a second rituximab course six months after cladribine if minimal residual disease (MRD) was detected in the blood. Lymphopenia and neutropenia are seen in 65-75% of patients by week two, which gradually improves by week four to six. The complete remission (CR) rate from the cladribine plus rituximab regime was 95%, and the median duration of CR was 70.1 months. The patients with TP53 mutations had a shorter progression-free survival (PFS) (median, 36.4 months vs. unreached; P = .0024) and OS (median, 52.4 months vs. unreached; P = .032). Similarly, subsets with MRD-negative CR at six months were showing a longer PFS (unreached vs. 17.4 months; P < .0001) and OS (unreached vs 38.2 months; P < .0001) [6]. Very limited data is available regarding the median overall survival of patients in CR and patients without CR for other cases or case series since no follow-up treatment details are available. 

Many treatment modalities have been attempted to treat HCLv with gradual evolution. Splenectomy is a potential treatment option shown in a study by Matutes et al. [7]. It showed remission in 13 out of 19 patients lasting one to ten years, with a median of four years. Pentostatin and α-interferon, active in HCLc, have shown poor responses in HCLv [1-4,8]. Allogenic marrow transplantation has been tried in a patient with HCLv, which achieved clinical remission for only 16 months [9]. Splenic irradiation and alemtuzumab have also been attempted in HCLv with limited success [10,11]. The bendamustine plus rituximab combination was attempted in three cases with a good response in first-line treatment in HCLv [12]. Small studies showed the effect of the administration of cladribine and late rituximab after one month of cladribine [13]. It is debatable whether starting rituximab concurrent with cladribine or after one month is preferable. There is a possibility of infections and fever with cladribine infusion, which makes concurrent rituximab administration difficult.

Due to the rarity of this disease and the limited number of studies, no clear second or subsequent lines of therapy are superior to retreatment with cladribine or any other choice. Once the patient relapses, several second-line options are rituximab, a monoclonal antibody against CD52 (alemtuzumab), and combination chemotherapies such as CHOP or CHOP-R [1-4,11]. Newer therapies may include treatment with a recombinant immunotoxin with an anti-CD22 variable domain fused to a truncated Pseudomonas endotoxin, moxetumomab pasudotox, a Bruton’s tyrosine kinase inhibitor, ibrutinib, and mitogen-activated protein kinase (MEK) inhibitors if MAP2K1 mutation is present [1-4]. 

The rearrangements expressing immunoglobulin variable heavy chain gene, VH4-34, commonly used in autoimmune disorders, were found in 40% of patients with HCLv versus 10% with HCLc in a study by Arons et al. [14]. VH4-34+ patients were found to have higher white blood cell counts at diagnosis, a lower response rate and PFS, and shorter overall survival with first-line cladribine [14]. 

The cases of HCLv are scarce, leading to the publication of a limited number of case reports and small series. An extensive search for the concerned cases was done on the internet, and 28 case reports were found from 1996 to 2023, summarized in tabular form (Table 3) [3,4,9-12,15-36]. There are no phase 3 studies to date due to rare occurrences. Limited phase 2 studies and a series of cases are summarized in tabular form (Table 4) [6,8,13,37-42]. 

**Table 3:** Published case reports of HCLv

Author	Year	Country	Topic	Age/Sex
Sgarabotto et al. [10]	1996	Italy	Remission in Hairy Cell Leukemia-Variant Following Splenic Radiotherapy Alone	79/M
Palomera et al. [15]	2002	Spain	Cladribine (2-chlorodeoxyadenosine) therapy in hairy cell leukemia variant. A report of three cases	NA
Goldaniga et al. [16]	2004	Italy	Clinical and molecular complete remission in a case of variant hairy cell leukemia treated with DHAP followed by high-dose chemotherapy plus rituximab	NA
Quach et al. [17]	2005	Australia	Complete remission of hairy cell leukemia variant (HCL-v) complicated by red cell aplasia post treatment with rituximab	NA
Narat et al. [18]	2005	UK	Successful treatment of hairy cell leukemia variant with rituximab	53/M
Ya-In et al. [19]	2005	Canada	Hairy Cell Leukemia Variant With Features of Intrasinusoidal Bone Marrow Involvement	68/F
Gupta et al. [20]	2005	India	Hairy cell leukemia-variant--a case report	66/M
Telek et al. [21]	2007	Hungary	Successful alemtuzumab treatment of a patient with atypical hairy cell leukaemia variant	58/M
Hadzi-Pecova et al. [22]	2008	Republic of Macedonia	Rituximab in the treatment of the variant of hairy cell leukaemia: a case report	57/F
Sasaki et al. [11]	2008	Japan	Effective Treatment of a Refractory Hairy Cell Leukemia Variant with Splenic Pre-Irradiation and Alemtuzumab	72/M
Busemann et al. [9]	2010	Germany	Late extramedullary relapse after allogeneic transplantation in a case of variant hairy cell leukaemia	60/M
Hsieh et al. [23]	2011	Taiwan	Hairy cell leukemia and variant in Taiwan: report of a variant case and literature review	67/M
Kanellis et al. [24]	2011	Greece	Hairy cell leukemia variant A description of the spleen morphology and immunophenotype of an archetypical case	79/M
Pande et al. [25]	2013	India	A Hairy Cell Leukaemia Variant – A Rare Case Report	58/F
Amelia et al. [26]	2015	Romania	A case of hairy cell leukemia variant	52/M
Rudolf-Oliveira et al. [27]	2015	Brazil	Hairy cell leukemia variant: the importance of differential diagnosis	74/M
Jian et al. [28]	2016	Canada	A Unique Hairy Cell Leukemia Variant	65/F
Kapoor et al. [29]	2017	India	Hairy Cell Leukemia – Variant (HCL-V): A Separate Entity	65/F
Visentin et al. [12]	2017	Italy	Bendamustine plus rituximab is an effective first-line treatment in hairy cell leukemia variant: a report of three cases	77/F, 83/F, 90/M
McKay et al. [30]	2017	Australia	Hairy cell leukaemia variant with periarticular joint infiltration and excellent radiotherapy response	73/M
Bohn et al. [31]	2017	Austria	Ibrutinib for relapsed refractory hairy cell leukemia variant	82/M
Jain et al. [32]	2018	USA	Biclonal IGHV-4-34 hairy cell leukemia variant and CLL - successful treatment with ibrutinib and venetoclax	79/M
Andritsos et al. [33]	2018	USA	Trametinib for the treatment of IGHV4-34, MAP2K1-mutant variant hairy cell leukemia	52/M
Wiber et al. [34]	2019	France	Variant form of hairy cell leukemia	64, 72/M
Visentin et al. [3]	2020	Italy	Ibrutinib in relapsed hairy cell leukemia variant: A case report and review of the literature	48/F, 90/F
Otieno et al. [4]	2022	USA	Remarkable Response of Hairy Cell Leukemia Variant to Single-Agent Cladribine	68Y/M
Passucci et al. [35]	2022	Italy	High Curative Potential of Ibrutinib in Hairy Cell Leukemia Variant Refractory to Conventional Chemotherapy: A Case Report	62/F
Khan et al. [36]	2023	India	A Case of Variant Hairy Cell Leukaemia in Durable Complete Remission on Combination Therapy	50Y/F

**Table 4:** Published larger case series and studies related to HCLv

Author	Year	Country	Topic	Number of cases
Sainati et al. [8]	1990	UK	A Variant Form of Hairy Cell Leukemia Resistant to α-Interferon: Clinical and Phenotypic Characteristics of 17 Patients	17
Zinzani et al. [37]	1990	Italy	Hairy cell leukemia variant: a morphologic, immunologic and clinical study of 7 cases.	7
Robak et al. [38]	1999	Poland	2-chlorodeoxyadenosine (cladribine) in the treatment of hairy cell leukemia and hairy cell leukemia variant: 7-year experience in Poland	6
Ravandi et al. [13]	2011	USA	Phase 2 study of cladribine followed by rituximab in patients with hairy cell leukemia	5
Kreitman et al. [39]	2013	USA	Cladribine with Immediate Rituximab for the Treatment of Patients with Variant Hairy Cell Leukemia	10
Angelova et al. [40]	2018	USA	Clinicopathologic and molecular features in hairy cell leukemia variant:single institutional experience	23
Chihara et al. [6]	2021	USA	Long term follow-up of a phase II study of cladribine with concurrent rituximab with hairy cell leukemia variant	20
Rogers et al. [41]	2021	USA	Phase 2 study of ibrutinib in classic and variant hairy cell leukemia	7
Wei et al. [42]	2023	China	Clinical and molecular characteristics and prognosis of classical hairy cell leukemia and hairy cell leukemia variant	9

MRD measurements can be done with IHC, multicolor flow cytometry, or PCR methods. Our case showed a discrepancy in residual disease with 0.5% abnormal cells in bone marrow flow cytometry. However, no abnormal population of cells in flow cytometry was done on peripheral blood six months after therapy with cladribine and rituximab. Bone marrow examination is required to document remission post-treatment, and so it was performed, and flow was sent on the bone marrow sample. Flow cytometry or polymerase chain reaction on bone marrow is a better modality to detect MRD or early relapse in leukemias. We also wanted to check the residual disease status on blood as the largest study on HCLv by Chihara et al. had done flow on blood for remission status post-treatment [6]. So, in our case, this discrepancy was picked up where bone marrow showed 0.5% residual cells while peripheral blood showed no residual cells. There is no consensus in the cases where the MRD discrepancy is noted. We have advised a second course of Rituximab to our case as bone marrow showed 0.5% residual disease and is presumed to be an early site of relapse even before the obvious signs of failure in peripheral blood. There are no standard comparisons for various methods of MRD assessment [43]. There is no role of second rituximab in MRD-negative cases as of now, as MRD-negative patients were excluded from further doses of rituximab in the phase II study. Since HCLv differs from HCLc, we cannot use the same guidelines for both diseases. There are many dilemmas for a clinician which are yet to be answered. 

Which one is preferred out of concurrent and late rituximab with cladribine infusion? This also needs further research and consensus on whether to include rituximab maintenance in MRD-negative patients. Since it is an aggressive, poorly responsive disease, rituximab is a well-tolerated drug. Theoretically, there is no harm in adding rituximab maintenance in such cases to compensate for the poor disease biology. The role of adding Ibrutinib over second rituximab in MRD-positive cases at six months after cladribine and rituximab therapy needs further research and consensus. In MRD discrepant cases in bone marrow and blood, like in our case, what is ideal treatment is yet to be answered. There is no consensus on the preferred second-line therapy. To conclude, there are many unmet needs in the treatment part of HCLv, which warrants further research. 

## Conclusions

This case report summarizes the related scarce literature in one place and will likely help clinicians in decision-making and literature review for future cases. This case also highlights and replicates the poor response to the standard cladribine plus rituximab regime in such cases. This case reinforces the unmet need for future research in therapeutics for this aggressive disease. The role of MRD positivity, the timing of testing, the method of testing, and treatment implications are not standard at present, and this case reinforces the need for a clearer picture. 

## References

[1] Paillassa J, Safa F, Troussard X (2022). Updates in hairy cell leukemia (HCL) and variant-type HCL (HCL-V): rationale for targeted treatments with a focus on ibrutinib. Ther Adv Hematol.

[2] Liu Q, Harris N, Epperla N, Andritsos LA (2021). Current and emerging therapeutic options for hairy cell leukemia variant. Onco Targets Ther.

[3] Visentin A, Imbergamo S, Trimarco V (2020). Ibrutinib in relapsed hairy cell leukemia variant: a case report and review of the literature. Hematol Oncol.

[4] Otieno SB, Ogbeide O (2022). Remarkable response of hairy cell leukemia variant to single-agent cladribine. Cureus.

[5] Cawley JC, Burns GF, Hayhoe FG (1980). A chronic lymphoproliferative disorder with distinctive features: a distinct variant of hairy-cell leukaemia. Leuk Res.

[6] Chihara D, Arons E, Stetler-Stevenson M (2021). Long term follow-up of a phase II study of cladribine with concurrent rituximab with hairy cell leukemia variant. Blood Adv.

[7] Matutes E, Wotherspoon A, Brito-Babapulle V, Catovsky D (2001). The natural history and clinico-pathological features of the variant form of hairy cell leukemia. Leukemia.

[8] Sainati L, Matutes E, Mulligan S, de Oliveira MP, Rani S, Lampert IA, Catovsky D (1990). A variant form of hairy cell leukemia resistant to alpha-interferon: clinical and phenotypic characteristics of 17 patients. Blood.

[9] Busemann C, Schüler F, Krüger W, Kiefer T, Wuppermann M, Androshchuk M, Dölken G (2010). Late extramedullary relapse after allogeneic transplantation in a case of variant hairy cell leukaemia. Bone Marrow Transplant.

[10] Sgarabotto D, Vianello F, Radossi P (1997). Remission in hairy cell leukemia-variant following splenic radiotherapy alone. Leuk Lymphoma.

[11] Sasaki M, Sugimoto K, Mori T, Karasawa K, Oshimi K (2008). Effective treatment of a refractory hairy cell leukemia variant with splenic pre-irradiation and alemtuzumab. Acta Haematol.

[12] Visentin A, Imbergamo S, Frezzato F (2017). Bendamustine plus rituximab is an effective first-line treatment in hairy cell leukemia variant: a report of three cases. Oncotarget.

[13] Ravandi F, O'Brien S, Jorgensen J (2011). Phase 2 study of cladribine followed by rituximab in patients with hairy cell leukemia. Blood.

[14] Arons E, Kreitman RJ (2011). Molecular variant of hairy cell leukemia with poor prognosis. Leuk Lymphoma.

[15] Palomera L, Domingo JM, Sola C, Azaceta G, Calvo MT, Gutierrez M (2002). Cladribine (2-chlorodeoxyadenosine) therapy in hairy cell leukemia variant. A report of three cases. Haematologica.

[16] Goldaniga M, Guffanti A, Gianelli U, Magni M, Deliliers GL, Baldini L (2004). Clinical and molecular complete remission in a case of variant hairy cell leukemia treated with DHAP followed by high-dose chemotherapy plus rituximab. Haematologica.

[17] Quach H, Januszewicz H, Westerman D (2005). Complete remission of hairy cell leukemia variant (HCL-v) complicated by red cell aplasia post treatment with rituximab. Haematologica.

[18] Narat S, Gandla J, Dogan A, Mehta A (2005). Successful treatment of hairy cell leukemia variant with rituximab. Leuk Lymphoma.

[19] Ya-In C, Brandwein J, Pantalony D, Chang H (2005). Hairy cell leukemia variant with features of intrasinusoidal bone marrow involvement. Arch Pathol Lab Med.

[20] Gupta K, Jasmina A, Malhotra P (2005). Hairy cell leukemia-variant--a case report. Indian J Pathol Microbiol.

[21] Telek B, Batár P, Udvardy M (2007). Successful alemtuzumab treatment of a patient with atypical hairy cell leukaemia variant (Artice in Hungarian). Orv Hetil.

[22] Hadzi-Pecova L, Stojanovik A, Petrusevska G, Panovska I (2008). Rituximab in the treatment of the variant of hairy cell leukaemia: a case report. Prilozi.

[23] Hsieh YC, Chang ST, Chuang SS, Lu CL, Tsao CJ, Lin CN, Li CY (2011). Hairy cell leukemia and variant in Taiwan: report of a variant case and literature review. Int J Clin Exp Pathol.

[24] Kanellis G, Garcia-Alonso L, Camacho FI (2011). Hairy cell leukemia variant. J Hematopathol.

[25] Pande P, Yelikar BR, Kumar U M (2013). A hairy cell leukaemia variant - a rare case report. J Clin Diagn Res.

[26] Amelia MG, Camelia D, Mihnea AG (2015). A case of hairy cell leukemia variant. Rom J Morphol Embryol.

[27] Rudolf-Oliveira RC, Pirolli MM, de Souza FS, Michels J, Santos-Silva MC (2015). Hairy cell leukemia variant: the importance of differential diagnosis. Rev Bras Hematol Hemoter.

[28] Jian C, Hsia CC (2016). A unique hairy cell leukemia variant. Case Rep Oncol.

[29] Kapoor AK, Kamal J, Singh A, Singh P (2017). Hairy cell leukemia - variant (HCL-V): a separate entity. J Med Sci Clin Res.

[30] McKay MJ, Rady KL, McKay TA (2017). Hairy cell leukaemia variant with periarticular joint infiltration and excellent radiotherapy response. Ann Transl Med.

[31] Bohn JP, Wanner D, Steurer M (2017). Ibrutinib for relapsed refractory hairy cell leukemia variant. Leuk Lymphoma.

[32] Jain P, Kanagal-Shamanna R, Konoplev S, Zuo Z, Estrov Z (2018). Biclonal IGHV-4-34 hairy cell leukemia variant and CLL - successful treatment with ibrutinib and venetoclax. Am J Hematol.

[33] Andritsos LA, Grieselhuber NR, Anghelina M (2018). Trametinib for the treatment of IGHV4-34, MAP2K1-mutant variant hairy cell leukemia. Leuk Lymphoma.

[34] Wiber M, Maitre E, Cornet E, Salaün V, Naguib D, Troussard X (2019). Variant form of hairy cell leukemia. Clin Case Rep.

[35] Passucci M, Ligia S, Giudice I (2022). High curative potential of ibrutinib in hairy cell leukemia variant refractory to conventional chemotherapy: a case report. Hematol Oncol Curr Res.

[36] Khan A, Krishna V, Reddy DN (2023). A case of variant hairy cell leukaemia in durable complete remission on combination therapy. Clin Oncol Case Rep.

[37] Zinzani PL, Lauria F, Buzzi M (1990). Hairy cell leukemia variant: a morphologic, immunologic and clinical study of 7 cases. Haematologica.

[38] Robak T, Błasińska-Morawiec M, Błoński J (1999). 2-Chlorodeoxyadenosine (cladribine) in the treatment of hairy cell leukemia and hairy cell leukemia variant: 7-year experience in Poland. Eur J Haematol.

[39] Kreitman RJ, Wilson W, Calvo KR (2013). Cladribine with immediate rituximab for the treatment of patients with variant hairy cell leukemia. Clin Cancer Res.

[40] Angelova EA, Medeiros LJ, Wang W (2018). Clinicopathologic and molecular features in hairy cell leukemia-variant: single institutional experience. Mod Pathol.

[41] Rogers KA, Andritsos LA, Wei L (2021). Phase 2 study of ibrutinib in classic and variant hairy cell leukemia. Blood.

[42] Wei C, Jin XX, Cai H, Wang X, Zhuang JL, Zhou DB (2023). Clinical and molecular characteristics and prognosis of classical hairy cell leukemia and hairy cell leukemia variant (Article in Chinese). Zhonghua Nei Ke Za Zhi.

[43] Ravandi F, Kreitman RJ, Tiacci E (2022). Consensus opinion from an international group of experts on measurable residual disease in hairy cell leukemia. Blood Cancer J.

